# Editors’ page

**DOI:** 10.21542/gcsp.2017.16

**Published:** 2017-10-31

**Authors:** Magdi H. Yacoub, Robert O. Bonow

**Affiliations:** 1 Imperial College, London, UK; 2 Feinberg School of Medicine, Northwestern University, Chicago, USA

This issue of the Journal, devoted largely to a single topic, is an attempt by the Journal to evolve its policies to meet the needs of our readers.

Kawasaki disease is conceived to be neglected, in spite of its global burden. The disease has been compared to the equally neglected, but commoner, rheumatic heart disease (RHD), which was recently the focus of a dedicated meeting in Cairo, entitled “Rheumatic Heart Disease - from Molecules to the Community”, and resulted in some specific recommendations known as “the Cairo Accord”^1^.

It is hoped that the current issue, edited by two experts in Kawasaki disease, will stimulate the evolution of concerted efforts to understand and possibly eradicate this important disease.

We  welcome the views of our readers both in terms of the concept of special issues, as well as topics for future such issues. 
